# “Hypertension is such a difficult disease to manage”: federally qualified health center staff- and leadership-perceived readiness to implement a technology-facilitated team-based hypertension model

**DOI:** 10.1186/s43058-024-00587-8

**Published:** 2024-05-02

**Authors:** Cristina Gago, Elaine De Leon, Soumik Mandal, Franze de la Calle, Masiel Garcia, Doreen Colella, Isaac Dapkins, Antoinette Schoenthaler

**Affiliations:** 1 Institute for Excellence in Health Equity, NYU Langone Health, 180 Madison Avenue, 7th Floor, New York, NY 10016 USA; 2 Family Health Centers at NYU Langone, Brooklyn, NY USA

**Keywords:** Implementation science, Hypertension, Medication adherence, Health inequities, Minority health, Practice facilitation, Primary care, Federally qualified health centers, Mixed methods evaluation

## Abstract

**Background:**

Despite decades of evidence demonstrating the efficacy of hypertension care delivery in reducing morbidity and mortality, a majority of hypertension cases remain uncontrolled. There is an urgent need to elucidate and address multilevel facilitators and barriers clinical staff face in delivering evidence-based hypertension care, patients face in accessing it, and clinical systems face in sustaining it. Through a rigorous pre-implementation evaluation, we aimed to identify facilitators and barriers bearing the potential to affect the planned implementation of a multilevel technology-facilitated hypertension management trial across six primary care sites in a large federally qualified health center (FQHC) in New York City.

**Methods:**

During a dedicated pre-implementation period (3–9 months/site, 2021–2022), a capacity assessment was conducted by trained practice facilitators, including (1) online anonymous surveys (*n* = 124; 70.5% of eligible), (2) hypertension training analytics (*n* = 69; 94.5% of assigned), and (3) audio-recorded semi-structured interviews (*n* = 67; 48.6% of eligible) with FQHC leadership and staff. Surveys measured staff sociodemographic characteristics, adaptive reserve, evidence-based practice attitudes, and implementation leadership scores via validated scales. Training analytics, derived from end-of-course quizzes, included mean score and number attempts needed to pass. Interviews assessed staff-reported facilitators and barriers to current hypertension care delivery and uptake; following audio transcription, trained qualitative researchers employed a deductive coding approach, informed by the Consolidated Framework for Implementation Research (CFIR).

**Results:**

Most survey respondents reported moderate adaptive reserve (mean = 0.7, range = 0–1), evidence-based practice attitudes (mean = 2.7, range = 0–4), and implementation leadership (mean = 2.5, range = 0–4). Most staff passed training courses on first attempt and demonstrated high scores (means > 80%). Findings from interviews identified potential facilitators and barriers to implementation; specifically, staff reported that complex barriers to hypertension care, control, and clinical communication exist; there is a recognized need to improve hypertension care; in-clinic challenges with digital tool access imposes workflow delays; and despite high patient loads, staff are motivated to provide high-quality cares.

**Conclusions:**

This study serves as one of the first to apply the CFIR to a rigorous pre-implementation evaluation within the understudied context of a FQHC and can serve as a model for similar trials seeking to identify and address contextual factors known to impact implementation success.

**Trial registration:**

ClinicalTrials.gov NCT03713515, date of registration: October 19, 2018.

**Supplementary Information:**

The online version contains supplementary material available at 10.1186/s43058-024-00587-8.

Contributions to the literature

- There is an urgent need to elucidate and address multilevel facilitators and barriers federally qualified health center (FQHC) staff, patients, and healthcare systems experience concerning access, delivery and sustainability of hypertension interventions.
- This study offers a reproducible model for pre-implementation evaluation, drawing on rich qualitative and quantitative data sources across six FQHC sites.
- Findings suggest that facilitators to implementation of an evidence- and team-based hypertension care model include staff members’ familiarity and empathy with patients’ barriers to care, interest in continued learning, and commitment to team-based health delivery models; barriers include limited staffing and technology infrastructure amidst high patient demand.

## Background

For decades, hypertension has persisted as a leading cause of preventable cardiovascular morbidity and mortality globally [1–5]. Although recent U.S. trends suggest hypertension awareness and treatment are improving [6, 7], rates of hypertension-related morbidity remain high: one-in-two U.S. adults carry the diagnosis and three-in-four cases present as uncontrolled [8]. The burden of hypertension-related morbidity and mortality weighs heaviest on health disparity populations [9–11], representing differential and suboptimal implementation of hypertension prevention, screening, and treatment interventions.

Multilevel barriers contributing to these disparities are well-documented [12–14]. For example, at the patient level, lack of insurance, transportation, time, and social support inhibit patients from accessing hypertension treatment [13, 15]. In the context of federally qualified health centers (FQHCs) especially, primary care providers (PCP) often face time limitations [16], issues with reimbursement [17], and a pull to prioritize competing demands and responsibilities before hypertension care [18, 19]. At a health system level, on the other hand, a lack of standardized training in evidence-based hypertension guidelines [19, 20] and poorly integrated clinical decision support tools impede quality care delivery [21].

Despite an abundance of literature that documents post-implementation reflections on challenges experienced in hypertension intervention delivery and uptake, literature reporting pre-implementation evaluation methods and capacity assessments remains limited [22, 23]. Without clear methods for measuring and acting on challenges identified in the pre-implementation phase, implementers are missing a critical opportunity to tailor and adapt implementation protocols to best align with the needs of communities served. Elucidating pre-implementation evaluation methods and summarizing key predictors of readiness prior to initiation of implementation is urgently necessary, as a means of hastening the translation of research to practice [24].

In response, our team of researchers, practitioners, and quality improvement specialists collaboratively developed a multilevel, technology-facilitated approach, known as Advancing Medication Adherence for Long-term Improvements in Hypertension through a Team-based Care Approach (ALTA), across six primary care sites in a large FQHC in Brooklyn, New York. Grounded in the Consolidated Framework for Implementation Science (CFIR) [25], the current paper presents findings of a rigorous, pre-implementation evaluation, aimed at identifying FQHC staff-anticipated facilitators and barriers to ALTA implementation. In so doing, we aim to inform best practices for hypertension intervention implementation and provide a feasible and reproducible model of pre-implementation evaluation methods in the context of community-based primary care clinics.

## Methods

### Trial design

The current manuscript describes the pre-implementation evaluation of ALTA: an trial designed to evaluate practice facilitation as a method to support the implementation fidelity of a multilevel intervention for improved medication adherence and hypertension control (NCT03713515, 2018–2023). As previously described [26], ALTA is a technology-facilitated, team care approach in which clinical staff – including front desk staff (patient service associates (PSAs)), medical assistants (MAs), nurses (RNs), and PCPs – work collaboratively to improve medication adherence and blood pressure control in patients receiving care within primary care sites. Following a stepped-wedge design [27], the trial spans four key measurement phases, including that of usual care (3–15 months), pre-implementation (3–9 months), implementation (12 months), and follow-up (6–18 months). During the implementation stage, ALTA involves a collaborative effort among administrative and clinical staff to identify patients with uncontrolled hypertension who are non-adherent with their medication regimen using electronic health record (EHR)-embedded tools; order remote blood pressure (BP) monitoring for continuous care; provide patient training in home BP monitoring and use of the patient portal (MyChart); and deliver health coaching for medication adherence and lifestyle modifications via a virtual high-risk clinic managed by RNs. Community health workers also assist patients by addressing technology-related barriers and unmet social needs. The current paper reports findings from the pre-implementation phase, during which trained ALTA practice facilitators conduct the CFIR-guided capacity assessment through validated surveys, training analytics from relevant courses, and semi-structured interviews prior to launching ALTA at the sites [28].

### Study setting

Researchers, in partnership with FQHC leadership, selected and invited six primary care sites within the Brooklyn-based FQHC affiliated with a large urban academic medical system to participate in the ALTA trial. These six sites are within one of largest FQHC networks in the country, which serves more than 100,000 patients per year (representing over 600,000 doctor’s visits) and has provided care for over 50 years to a thriving community of mostly immigrant patients residing in Sunset Park, Brooklyn, New York. The sites met the following eligibility criteria: (1) use of Epic EHR for at least 6 months; (2) no immediate plans for hypertension-related quality improvement initiatives; and (3) willingness to sign a memorandum of understanding and identify a practice champion to work with research staff. Trained practice facilitators collected pre-implementation data from the first six sites of ten anticipated (April 2021—October 2022). One of the seven total ambulatory sites within this FQHC system was not included in the trial due to funding limitations; however, this remaining ambulatory site does plan to implement the intervention in the future.

### Conceptual framework

The documented lag in translating evidence-based hypertension management to practice [29, 30] represents a suboptimal implementation outcome, driven by multilevel barriers faced by health systems and staff in efficiently delivering hypertension care [31]. Therefore, we saw this as a natural opportunity for the application of Implementation Science theory to practice. Specifically, we chose to ground our evaluation metrics and analysis in the CFIR [32, 33], as it is one of the most widely published Implementation Science determinant frameworks [34]. We drew from CFIR 2.0 [33] (vs. the original 2009 CFIR [32]), as the revisions offered by the CFIR 2.0 addressed gaps in original domains and better centered the domains around recipient needs and the social determinants of health faced by health disparity populations seen in a FQHC [33].

**Fig. 1:**
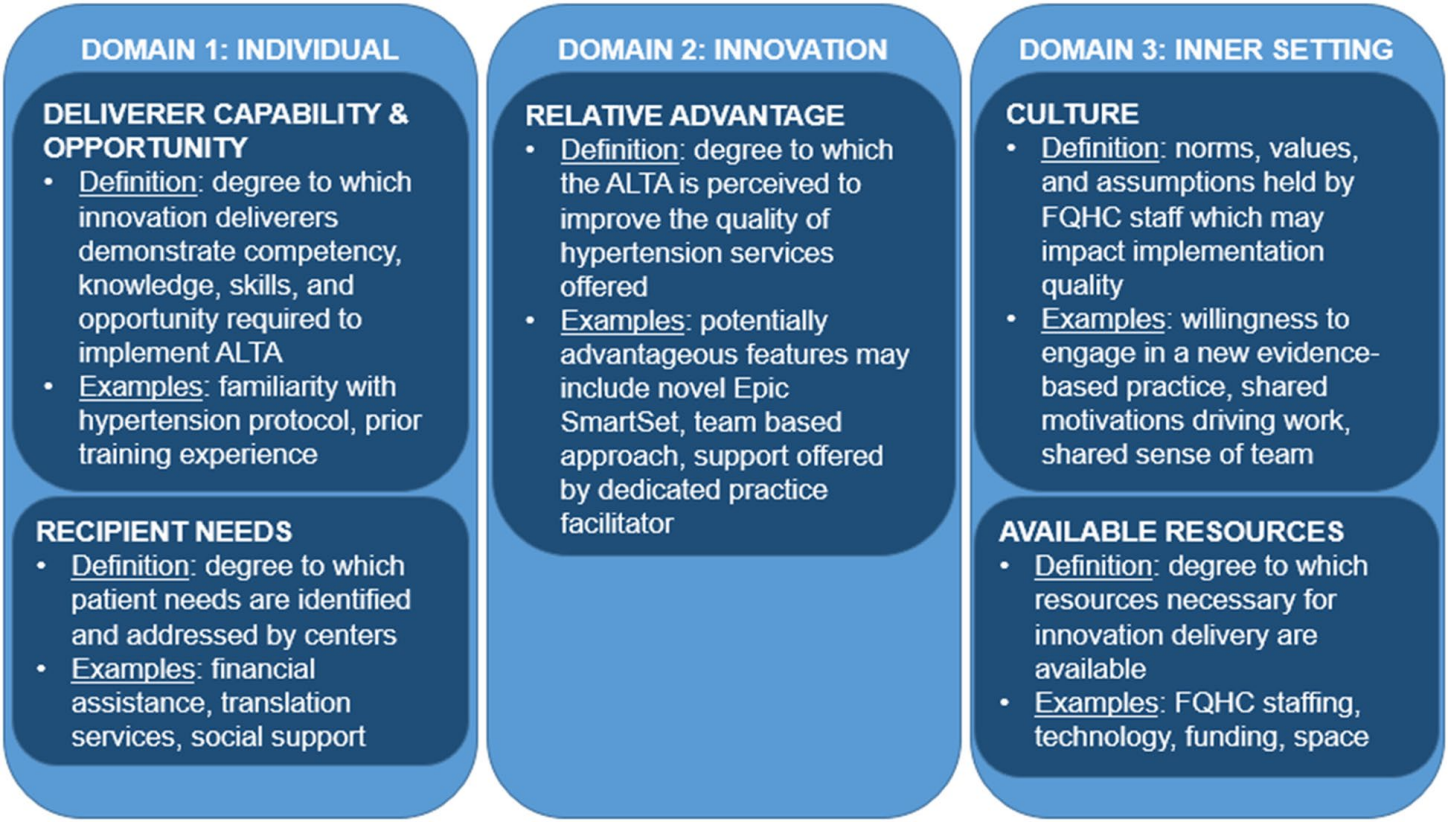
Conceptual framework for the current study informed by the Consolidated Framework for Implementation Research 2.0

Guided by the CFIR 2.0 (hereon described as “the CFIR”), this pre-implementation evaluation was designed to target three of five CFIR domains: the individual, innovation, and inner setting. The individual domain (constructs: deliverer capability and opportunity, recipient needs) captured characteristics of both ALTA implementers (FQHC staff) and intervention recipients (patients) which may affect ALTA intervention delivery and uptake, respectively. The innovation domain (construct: relative advantage) encompassed characteristics of ALTA, which may influence the degree of implementation success. Finally, the inner setting (constructs: available resources, culture) was characterized by the immediate environment in which ALTA would be implemented (e.g., the six sites). Together, these three domains were selected as we hypothesized, based on prior literature [12–14], they may capture known and novel facilitators and barriers to hypertension intervention implementation in the context of a FQHC (Fig. 1).

### Data collection

All data presented in the current paper was collected from FQHC leadership and staff (including PSAs, MAs, RNs, and PCPs) across the six participating sites, via surveys, training analytics, and semi-structured interviews during the pre-implementation stage (2021–2022). Table 1 summarizes the data sources (including measures, samples, and data) by target CFIR domain.

**Table 1:** Summary of data sources by the CFIR domain

**Measures**	**Sample**	**The CFIR Domains and Constructs ** [32]	**Definitions**	**Data**
**Survey**	• FQHC leadership (*n* = 18) • FQHC staff (*n* = 106)	**Individuals (deliverers): capability & opportunity**	Degree to which innovation deliverers demonstrate competency, knowledge, skills, and opportunity required to implement ALTA	• Adaptive reserve scores [35] • Evidence-based practice attitudes scores[36] • Implementation leadership scores [37]
**Training analytics**	• FQHC staff (*n* = 69)			• Mean number of attempts required to pass • Mean scores on first attempt
**Semi-structured interview**	• FQHC staff (*n* = 67)	**Individuals (recipients): needs**	Degree to which patient needs are identified and addressed by sites	• What are some topics you discuss with your patients with hypertension? • What type of support would patients need to upload their BP readings?
		**Innovation: relative advantage**	Degree to which the ALTA is perceived to improve the quality of hypertension services offered	• After going through the ALTA steps, what are your general thoughts about implementing a process like ALTA in your practice? • What are your suggestions for integrating the ALTA steps in your practice to improve the health outcomes of hypertensive patients?
		**Inner setting: culture**	Norms, values, and assumptions held by FQHC staff	• What is the process for measuring BP? Please walk me through each of the steps • How do you feel about taking a more team-based approach to managing patients with hypertension?
		**Inner Setting: available resources**	Degree to which resources necessary for innovation delivery are available (e.g., FQHC staff, technology, funding, space)	• What type of resources would help you support patients? • What kind of materials would be helpful for patients to have when they need to access MyChart away from the clinic?

#### Survey

Through a convenience sampling approach, all FQHC leadership and staff (*n* = 176 eligible) were invited over broadcast email to participate in a brief, anonymous online survey offered in English. Anonymous survey links were accessible via Research Electronic Data Capture (REDCap). Prior to completing the survey, every participant reviewed information about the study and entered the date as an indication of consent; those who completed the survey received a $25 gift card as incentive. The survey included questions on participant sociodemographic factors (e.g., gender, year of birth, race, ethnicity, and country of origin), employment experience (e.g., employment status, time with current site, and job title), and prior training (e.g., training in chronic disease management, patient-centered communication, and quality improvement methods). Three abbreviated scales were administered among staff and leadership, including the Adaptive Reserve Scale [35], Evidence-Based Practice Scale [36], and Implementation Leadership Scale [37] (Table 1), measuring constructs hypothesized to influence implementation fidelity [26]. The Adaptive Reserve Scale (*n* = 14 items) measured a site’s ability to make and sustain change. The Evidence-Based Practice Attitudes Scale (*n* = 11 items) measured attitudes regarding the appeal of, requirements of, openness towards, and divergence between evidence-based practice and usual care. The Implementation Leadership Scale (*n* = 8 items for staff and *n* = 9 for leadership) measured the degree to which leadership is proactive, knowledgeable, supportive, and perseverant.

#### Training analytics

FQHC leadership selected and assigned staff to complete online courses created by the ALTA team in accurate BP measurement in the office, self-measured BP (i.e., how patients should self-measure BP at home), and/or quality improvement, in line with evidence-based guidelines from the American Heart Association [7], Target: BP™ [38], and the Joint National Committee on Prevention, Detection, Evaluation, and Treatment of High Blood Pressure [39]. Multiple choice comprehension questions were asked following two trainings on accurate BP measurement in the office (*n* = 4 items) and self-measured BP (*n* = 6 items). FQHC staff were required to retake the survey as many times as was required to receive a passing score of 75% or higher.

#### Semi-structured interview

Development of the semi-structured interview guides was grounded in the CFIR [32] and prior literature on multi-level barriers and facilitators to implementation of hypertension care innovations [31]. All FQHC staff were eligible to participate in the semi-structured interviews, and interviewees were recruited via convenience sampling. Practice facilitators set up calls conducted over Webex video conference over several dates for each site and invited all FQHC staff to interview. Each interview took between 5–20 min. Practice facilitators conducted semi-structured interviews to understand each site’s existing hypertension management workflow, including needs and resources related to hypertension care as well as perceived fit and advantage of ALTA as a proposed incoming program. Semi-structured interviews were conducted in English, audio-recorded via Webex and transcribed *verbatim* by a professional third party. Consent was not required and incentives were not provided, as interviews were conducted for quality improvement purposes. Originally, 117 interviews were conducted across all sites (representing approximately 85% of eligible FQHC staff, *n* = 138). However, due to technology failure, 50 interviews were lost and unrecoverable in the process of an unexpected Webex update. The remaining 67 interviews (representing 48.6% of eligible FQHC staff) were included in the analysis (Table S1).

### Analysis

#### Survey

Survey responses were downloaded from REDCap as a CSV file for analysis. Only complete responses were included in the analysis (*n* = 124); partial responses were excluded (*n* = 24). For each scale (e.g., adaptive reserve, evidence-based practice, and implementation leadership) and subscale, overall mean (SD) scores are calculated and presented across all sites as well as the minimum and maximum scores across sites. Adaptive reserve scores are interpreted on a scale of 0–1, while evidence-based practice attitude and implementation leadership scores are interpreted on a scale of 0–4; higher scores are interpreted as advantageous for all scales. To protect the anonymity of individual sites, the minimum and maximum values are presented in aggregate, rather than unique results for the six sites. Survey data were downloaded from REDCap and analyzed using SAS 9.4 (North Carolina).

#### Training analytics

Training analytic data were downloaded from the FOCUS training platform as a CSV file for analysis. For trainings with comprehension checks (e.g., those covering accurate in-office and self-measured BP measurement), the mean (SD) number of attempts needed to pass and the mean score on first attempt (percent questions answered correct out of 100%) are reported, overall as well as minimum and maximum across sites. Training analytic data were downloaded from REDCap and analyzed using SAS 9.4 (North Carolina).

#### Semi-structured interview

Prior to analysis, CG and EDL deductively created a preliminary CFIR-grounded codebook, with codes representing CFIR constructs described above (deliverer capability and opportunity, recipient needs, relative advantage, available resources, and culture). After reviewing and discussing the codebook, trained qualitative coders (CG, EDL, and SM) met to code the first interview transcript. Next, after each of the coders independently coded the same ten interviews, the group met again to compare codes, ensure consistency in coding, and reconcile differences through discussion [40, 41]. After establishing that coding was consistent across all coders, the remaining 56 interviews were divided among the coders to code independently as single reviewers. Doubts and points of concern in coding were documented as memos and brought to the group for discussion in a third meeting, at which point, the coders reviewed all quotes under each code to collaboratively generate themes. This analysis was conducted across all transcripts and stratified by site to establish consistency of themes across data source and site. After themes were established, they were shared and discussed for member checking with the supervising ALTA practice facilitator, who had established relationships with staff across all sites, and confirmed alignment between themes and on-the-ground experience. All qualitative coding was conducted using Dedoose Version 9.0.17 (Los Angeles, CA) [42].

The trained qualitative researchers (CG, EDL, SM) who collaboratively and iteratively coded the transcripts acknowledge salient identities brought to their coding process, which impacted their interpretation of the data. CG is a white Spanish American woman, training as a postdoctoral fellow in population health, with an emphasis on program evaluation. ED is a Dominican American woman and physician scientist who practices primary care at one of the primary care sites included in the study. SM is a man of Asian descent and a postdoctoral researcher in population health, investigating digital health technologies from human–computer interaction lenses.

### Ethics approval and consent to participate

This study was approved by the Institutional Review Board of the New York University Langone Health (NYULH, reference number: s18-01290).

## Results

### Survey (the CFIR’s individual domain)

#### Sociodemographic and employment characteristics

A majority of FQHC leadership and staff eligible for the survey participated (*n* = 124 of 176 eligible; Table S1). Of those who responded, approximately one-in-four were under the age of 35, a majority female (83.1%), and approximately one third Latino/a or Hispanic (34.7%; Table 2). About half were born in a country other than the United States (45.2%) and have been in their current FQHC position for less than 3 years (48.4%). An equal number of respondents identified as a PCP (25.8%), MA (24.2%), or “other” (25.8%; e.g., PSA, RN).

**Table 2:** Sample characteristics of FQHC staff surveyed across six sites (2021–2022; Brooklyn, NY)

**Socio-demographics**	**All**	**Site 1**	**Site 2**	**Site 3**	**Site 4**	**Site 5**	**Site 6**
	(*n* = 124)	(*n* = 21)	(*n* = 10)	(*n* = 26)	(*n* = 20)	(*n* = 35)	(*n* = 12)
	**%**^a^	**%**^a^	**%**^a^	**%**^a^	**%**^a^	**%**^a^	**%**^a^
**Age in years**							
< 35	22.6	19.1	20.0	15.4	10.0	42.9	8.3
35–45	33.1	28.6	60.0	38.5	45.0	28.6	0.0
46–55	25.8	38.1	20.0	26.9	35.0	14.3	25.0
> 55	18.6	14.3	0.0^b^	19.2	10.0	14.3	66.7
**Gender**							
Male	16.9	9.5	30.0	7.7	10.0	28.6	16.7
Female	83.1	90.5	70.0	92.3	90.0	71.4	83.3
**Race & ethnicity**							
Black or African American	14.5	57.1	0.0^b^	15.4	0.0^b^	5.7	0.0^b^
Asian	15.3	0.0^b^	10.0	3.9	0.0^b^	25.7	66.7
Latino/a or Hispanic	34.7	9.5	60.0	50.0	50.0	28.6	16.7
Other	35.5	33.3	30.0	30.8	50.0	40.0	16.7
**Born**							
USA	54.8	38.1	70.0	57.7	60.0	62.9	33.3
Another Country	45.2	61.9	30.0	42.3	40.0	37.1	66.7
**Employment**							
**Role**^c^							
Leadership	14.5	23.8	30.0	11.5	15.0	8.6	8.3
PCP	25.8	14.3	30.0	11.5	20.0	45.7	25.0
RN	9.7	4.8	0.0^b^	11.5	15.0	11.4	8.3
MA	24.2	23.8	0.0^b^	30.8	25.0	17.1	50.0
Other (e.g., PSA)	25.8	33.3	40.0	34.6	25.0	17.1	8.3
**Years in current position**							
0–2	48.4	61.9	50.0	50.0	40.0	57.1	8.3
3–5	15.3	9.5	40.0	15.4	20.0	11.4	8.3
6–15	21.0	14.3	10.0	19.2	15.0	25.7	41.7
16 +	14.5	14.3	0.0^b^	15.4	20.0	5.7	41.7

^a^Column percent is reported overall and by site, for those who responded to the survey. Columns may not sum to 100% due to missingness

^b^Note that some cells may contain a value of “0.0” if no *survey respondent* represented that category

^c^*PCP* Primary care physician (including physicians, physicians assistants, nurse practitioners, and licensed practical nurses), *RN* Registered nurse, *MA* Medical assistant, and *PSA* Patient services advocate

#### Adaptive reserve

The mean (SD) adaptive reserve score across all FQHC leadership and staff respondents (*n* = 124) was moderately positive (0.7 (0.1); range = 0–1; Tables 3 and S2). This means that most staff positively rated their site's *ability to make and sustain change, like that of ALTA* (e.g., “People in our practice actively seek new ways to improve how we do things” and “Leadership strongly supports practice change efforts”). Further, variation in mean (SD) across sites was minimal, with the lowest score being 0.7 (0.2) vs. highest 0.8 (0.2), meaning that scores were consistent across sites.

**Table 3:** Mean adaptive reserve scores and frequency of item responses

**Summary scores**	**Overall**	**Minimum across sites**	**Maximum across sites**
	**Mean (SD)**^c^	**Mean (SD)**^c^	**Mean (SD)**^c^
**Adaptive reserve (range: 0–1)**^a^	**0.7 (0.1)**	**0.7 (0.2)**	**0.8 (0.2)**
**Evidence-based practice attitudes (range: 0–4)**^b^	**2.7 (0.7)**	**2.3 (0.7)**	**3.1 (0.7)**
Subscale 1: Openness	2.8 (0.9)	2.1 (1.1)	3.2 (0.8)
Subscale 2: Divergence (reverse)^d^	1.8 (1.0)	1.4 (0.9)	2.3 (0.8)
Subscale 3: Appeal	2.6 (0.9)	2.2 (0.9)	2.9 (0.8)
Subscale 4: Requirements	2.6 (1.0)	1.8 (0.9)	3.0 (0.8)
**Implementation leadership (range: 0–4)**^a^	**2.5 (0.9)**	**2.0 (1.1)**	**2.9 (0.9)**
Subscale 1: Knowledgeable	2.6 (1.0)	2.3 (0.5)	3.0 (1.1)
Subscale 2: Supportive	2.7 (1.0)	2.1 (1.2)	3.0 (1.0)
Subscale 3: Perseverant	2.6 (1.0)	2.0 (1.2)	2.9 (0.9)
Subscale 4: Proactive	2.4 (1.1)	1.9 (1.1)	2.7 (1.2)

^a^All FQHC leadership and staff (*n* = 176) were asked to complete the Adaptive Reserve Scale and the Implementation Leadership Scale; of those eligible, 70.4% (*n* = 124) completed the scale

^b^All FQHC staff (*n* = 153) were asked to complete the Evidence-Based Practice Attitudes Scale; of those eligible, 69.3% (*n* = 106) completed the scale

^c^Site-level mean (SD) score and frequencies are compared; both the minimum and the maximum observed across sites are presented

^d^Higher score is interpreted as disadvantageous, contrasting the interpretation of other scores

#### Evidence-based practice attitudes

The mean (SD) evidence-based practice attitudes score across all FQHC staff respondents (*n* = 106) was moderately positive (2.7 (0.7), range = 0–4; Tables 3 and S3), as were the subscores: *openness* to evidence-based practices (2.8 (0.9)), *appeal* of evidence-based practices (2.6 (0.9)), and likelihood of adopting practices given the *requirement* to do so (2.6 (1.0)). Further, reported *divergence* of usual from evidence-based practice was low (1.8 (1.0)), meaning that most staff did not see their usual practice as deviating from evidence-based standards. This means that, overall, most staff reported relatively positive attitudes toward adoption of new evidence-based practice, such as ALTA. For example, a majority of staff reported liking "to use new types of interventions to help my patients" and being "willing to use new and different types of interventions developed by researchers".

#### Implementation leadership

The mean (SD) implementation leadership score across all FQHC leadership and staff respondents (*n* = 124) were moderately positive (2.5 (0.9); range = 0–4; Table 3 and S4) with minimal variation by site (minimum = 2.0 (1.1), maximum = 2.9 (0.9)). This means that most staff found site leadership to be knowledgeable about evidence-based practices, supportive of evidence-based practice implementation, and perseverant in surmounting challenges in the implementation journey; however, just under 50% of respondents agreed that leadership "removed obstacles to the implementation of evidence-based practice for improved hypertension management" and " established clear standards for the implementation of evidence-based practice for improved hypertension management". Further, only one-in-ten leaders reported having developed a plan to facilitate implementation of evidence-based practice for hypertension management.

#### Prior training

Fewer than one-in-two FQHC staff reported having received prior training in chronic disease management (33.9%), patient-centered communication (39.5%), and quality improvement methods (46.0%, Table 4); however, responses varied across sites.

**Table 4:** Reported history of prior trainings and current training analytics

	**All**	**Minimum across sites**	**Maximum across sites**
**Frequency report prior training (*n* = 124)**^a^	**%**^e^	**%**^e^	**%**^e^
Chronic disease management	33.9	9.5	58.3
Patient-centered communication	39.5	19.2	58.3
Quality improvement methods	46.0	28.6	66.7
**Training 1: Accurate blood pressure measurement in office (*n* = 69)**^b^	**Mean (SD)**^f^		
Comprehension score, first attempt^c^	82.1 (20.9)		
Number attempts needed to pass^d^	1.4 (0.8)		
**Training 2: Self-measured blood pressure (*n* = 29)**^b^	**Mean (SD)**^f^		
Comprehension score, first attempt^c^	89.7 (9.4)		
Mean (SD) number attempts needed to pass^d^	1.4 (0.8)		

^a^Data presented was collected via online survey; all FQHC staff were eligible and invited to participate

^b^Data derived from online training module analytics

^c^Four comprehension questions were asked following Training 1 and six were asked following Training 2; based on responses, percent correct out of 100% were assigned. A score of 75% or higher was considered “passing”

^d^FQHC staff were required to complete the training comprehension quiz as many times as necessary to receive a score of 75% or higher; presented is the mean (SD) number of times a FQHC staff member was required to complete the training to receive a passing score

^e^The frequency of survey respondents (*n* = 124) who report prior training the relevant areas are reported

^f^Mean (SD) scores were not tabulated by site due to small sample size

### Training analytics (the CFIR’s individual domain)

A majority of FQHC staff assigned to three training courses completed them: 94.5% for accurate BP measurement in office, 90.6% for self-measured BP, and 90.3% quality improvement (Table S1). The mean (SD) comprehension score on first attempt was over the passing threshold of 75%. However, comprehension scores demonstrated high variability for the training on in-office accurate BP measurement (mean = 82.1%, SD = 20.9%), more so than for the self-measured BP (mean = 89.7%, SD = 9.4%; Table 4). The mean (SD) number of attempts needed to pass was the same for both trainings: between one and two attempts were needed (1.4 (0.8)).

### Semi-structured interview (the CFIR’s innovation, inner setting, and individual domains)

From the qualitative analysis, four key themes emerged, each aligning with a unique CFIR construct; illustrative quotations for each are included in Table 5. Specifically, staff reported that (1) complex barriers to hypertension care, control, and clinical communication exist; (2) there is a recognized need to improve hypertension care; (3) in-clinic challenges with digital tool access imposes workflow delays; and (4) despite high patient loads, FQHC staff are motivated to provide high-quality care. Responding to each of these potential facilitators and barriers to implementation, research staff proposed adaptive implementation remedies (Table 6).

**Table 5:** Illustrative quotations from semi-structured interviews with FQHC staff

**Theme 1 (the CFIR’s individual recipients: needs): complex extra-clinical barriers to hypertension care and control exist**	
Barriers to hypertension control	“Even though [the] provider will prescribe the blood pressure machine, [patients] can’t afford to buy it.” (Record 39, RN) “The big thing here I think is money for patients because a lot of them are not insured… if they have five medications [they will take the ones] that they think [are] most important…and then say, ‘Well, I will go pick the high blood pressure medication next week when I get some money’.” (Record 21, RN)
Digital access & literacy	“I know a lot of our patients here…don't even have MyChart or don't even know how to access it. I think it's just that's like the limiting factor. I think they're willing to do it. I think it's just if it's easy or not for them to do it.” (Record 61, PCP) “I think a lot of patients are capable, but… some of the patients are struggling with the telemedicine video visit … [and] a lot of people need [assistance]… Number two, they need that device, they need that internet" (Record 25, PCP)
**Theme 2 (the CFIR’s innovation: relative advantage): FQHC staff recognize the need to improve hypertension care**	
Urgency to address hypertension	“I actually feel and believe that [ALTA] is really needed. I think it will be a very helpful program not only [for] the patients, [but] also for us to improve.” (Record 22, MA) “I think it's great, I think our population would definitely, definitely benefit… [and] hopefully we would see better indications of our patients actually taking good care of their health.” (Record 39, RN)
Hypertension workflow *varies by site*	“We had some programs many years ago but that can be about 20 to 30 years.” (Record 22, MA) “[Hypertension protocol is] pretty standard across the board.” (Record 14, MA)
**Theme 3 (the CFIR’s inner setting: available resources): in-clinic challenges with digital tool access imposes workflow delays**	
Limited internet connectivity and access to technology	“The reception is not that good and people do not like to use LTEs, so …most of them are not able to download an app while they are here [because] the internet is not strong enough.” (Record 53, PCP) “Another thing too what makes it sometimes difficult for us to enroll them is that the signal here at this location is very weak… [and] when we do try to assist them, we tell our patients, ‘Sorry you can't upload it to your MyChart app because of the [lack of] signal here at the clinic.” (Record 18, PCP)
Technical support demands staff time	“We could probably say let me take your phone and I'll help you… [but] we might not have the time available to dedicate… to help them sign up. So, we give them the information… [and] try our best to help them as much as we can.” (Record 50, PSA) "[It helps if patients are] able to come back and see how to [use MyChart] again… they must really bring the phone and see how the nurses does it in front of them." (Record 43, PCP)
**Theme 4 (the CFIR’s inner setting: culture): despite high patient loads, FQHC staff are motivated to provide high-quality care**	
Recipient-centeredness	“We want to improve things for [the patient] and teach them and guide them and [let them know] that we're here for [them].” (Record 32, PSA) “But the effort is there a 1,000%, and [we try] to make them feel comfortable. You want everybody's visit to be the best that it could be and offer them the most that you could give them.” (Record 13, PSA)
Deliverer-centeredness	“We always have to protect the patient and we have to protect ourselves too…Everybody needs… a team.” (Record 47, MA) “Well, if I had a magic wand, basically yeah, having more FQHC staff would be great. Like magic wand, everybody has their own doctor.” (Record 15, PSA)
Learning-centeredness *varies by site*	“We get evaluated every year, and every year we get different training… We sit in the conference when they are doing it, so we listen in, and do different types of comprehensive in-hand training." (Record 23, MA) “Specific training? We don't have a specific training." (Record 14, MA)

**Table 6:** Summary of FQHC staff-anticipated facilitators and barriers to upcoming hypertension trial implementation

**The CFIR domain**	**The CFIR construct**	**Potential facilitators to implementation**	**Potential barriers to implementation**	**Proposed implementation remedies**
Individual	Deliverer capability & opportunity	• Moderate adaptive reserve, evidence-based practice, and implementation leadership scores • High rates of training completion and high mean first-attempt scores	• Variable evidence-based practice scores across sites • Limited prior training in chronic disease management, patient-centered communication, and quality improvement methods	• Tailor implementation supports to match site-specific assets and needs • Offer accessible training opportunities in the areas FQHC staff report prior limited experience
	Recipient needs	• FQHC staff recognize and empathize with diverse patient needs	• Diverse barriers to accessibility of hypertension exist • Limited digital access and literacy among patients	• Provide patients with resources, training, and hands on support for connecting with and utilizing MyChart and related digital tools
Innovation	Relative advantage	• FQHC staff recognize a need for quality hypertension care which increases patient awareness • Hypertension workflows have been developed and rolled out across all sites	• Acknowledgement of and adherence to hypertension workflows varies by site • Presence of a preexisting hypertension program associated with limited perceived relative advantage offered by ALTA	• Assign relevant online training modules describing workflows and assesses comprehension via online quizzes • Practice facilitators may emphasize the distinction between preexisting and ALTA workflows and communicate the relative advantage ALTA offers
Inner Setting	Available resources	• In-clinic technology streamlines workflow	• Equipment availability is limited • Connectivity-related delays are common • Degree of technical supports offered by clinical FQHC staff varies across sites	• Invest in digital infrastructure, by equipping clinics with physical equipment, Wi-Fi, and space necessary to facilitate patient-provider communications
	Culture	• FQHC staff demonstrate a clear desire to connect with and assist patients • Adequate FQHC staff and time protect FQHC staff against stress and burnout	• FQHC staff perceived form and frequency of evaluation and feedback offered varies by site	• Practice facilitators provide standardized supervision, evaluation, and training to staff in the pre-implementation and early implementation stages

### Theme 1 (the CFIR’s individual recipient needs): complex barriers to hypertension care, control, and clinical communication exist.

The CFIR's individual needs construct is defined as the degree to which patient needs are recognized, identified, and addressed by FQHC sites. In line with this construct, Theme 1 elucidates the ways in which FQHC staff perceive, recognize, and acknowledge patient (or innovation "recipient") needs. Specifically, as described below, staff spoke to (1) the complex socio-environmental barriers patients face in accessing hypertension care and maintaining hypertension control, and (2) how patients’ limitations in digital access and literacy affect engagement with clinical communication and tools.

#### Subtheme 1.1: Barriers to hypertension control

FQHC staff emphasized that most hypertension-related patient behaviors occurred outside the clinic, with clinical encounters acting as a cross-sectional snapshot between extended periods apart between patients and their providers. Named barriers to hypertension self-management included: the (1) prohibitive cost of medication and/or BP monitors, (2) lack of social support in navigating social and clinical services, (3) presence and interaction of competing priorities (e.g., finances, housing, employment, food insecurity), and (4) common misperceptions regarding hypertension and treatment (e.g., hypertension can be “felt”). Staff also reported they believe patients are not forthcoming about their medication nonadherence, which undermines FQHC staff from forming the best treatment plan.

#### Subtheme 1.2: Digital access & literacy

Staff also identified patients' limitations in digital access and literacy as potential barriers to engagement with clinical communication tools (e.g., the patient portal, MyChart), which are necessary for engagement with the ALTA program. Common barriers to MyChart use among patients included (1) username and password issues, (2) lack of access to a smartphone or computer, (3) lack of familiarity or comfort using mobile applications, (4) limited understanding of what MyChart offers, and (4) preference for other forms of communication (e.g., phone call or face-to-face). Staff perceived engagement with MyChart to vary by age, with younger (vs. older) patients demonstrating greater willingness to use the platform, due to perceived differences in digital literacy (e.g., comfort using application) and access (e.g., owning a smartphone or computer). However, one of the most powerful facilitators to MyChart engagement was the presence of social support within a patients' life, in the form of children or relatives who were available to support navigation on the platform.

### Theme 2 (the CFIR’s innovation: relative advantage): FQHC staff recognize the need to improve hypertension care

The CFIR's relative advantage construct, embedded within the innovation domain, is defined as the degree to which ALTA, above and beyond usual care, is perceived to improve the quality of hypertension services offered. Reflective of this construct, Theme 2 encompasses staff reflections regarding (1) the perceived need and urgency for novel hypertension management programs, as well as (2) the perceived utility of a novel program like ALTA within the context of preexisting hypertension workflows.

#### Subtheme 2.1: Urgency to address hypertension

FQHC staff agreed that improvements in hypertension care are critically important, especially those that increase patient awareness and self-management of their BP, which staff described as limited. Though staff recognized the need for improved hypertension care and the importance of quality hypertension care, staff reported that patients' do not share that same sense of urgency, potentially due to misconceptions regarding risks associated with uncontrolled hypertension. In this regard, many staff expressed enthusiasm for a program like ALTA, which they believed may help to better fill critical gaps patient awareness regarding the risks of uncontrolled hypertension.

#### Subtheme 2.2: Hypertension workflow varies by site

While most staff stated they followed a BP measurement protocol, the degree to which protocols were acknowledged and adhered to varied by site. The hypertension clinical pathway and opportunities offered for additional training varied by site, with some sites having offered, for example, training and/or feedback through supervision, while staff from others recalled no hypertension-specific trainings in place. The source of the protocols also differed, with some staff reporting they learned the protocol at the site vs. others still followed the protocol they learned in their initial degree or certificate program. This demonstrated variability in standardization, mapped onto variability in perceived value of the ALTA program. For example, sites with a strong hypertension program did not perceive a need for additional services as compared to those lacking standard hypertension protocols.

### Theme 3 (the CFIR’s inner setting: available resources): in-clinic challenges with digital tool access imposes workflow delays

The CFIR's available resources construct, within the inner setting domain, is defined as the degree to which resources necessary for innovation (e.g., ALTA) delivery (e.g., staffing, time, space, funding) are available. Consequently, this theme speaks to staff reflections on how limitations in technology equipment and connectivity-related delays hinder workflows, as well as how patient demands for technical support pose significant burden FQHC staff time and effort, particularly for those in administrative roles.

#### Subtheme 3.1: Internet connectivity and access to technology impact interactions between staff and patients

ALTA interventions require patient access to MyChart (the EPIC patient portal). Although patients were able to access MyChart through their personal devices, many still required technical assistance (e.g., password recovery and email access). For those patients requiring assistance activating MyChart, availability of hardware (e.g., tablets) that could be used to activate MyChart in the clinic was a limiting factor. The practice makes a small number of tablets available to patients to assist in accessing MyChart while in the facility. Tablets that malfunctioned were, at times, a cause of bottlenecks in workflow. Lack of Wi-Fi connectivity and poor cellular reception in the buildings contributed to connectivity-related delays, adding additional time to patient encounters and consuming staff time. When patients were unable to enroll in MyChart at the clinic, they were unlikely to do it at home, where they lack hands on assistance from trained FQHC staff. ALTA clinical workflow dependence on real-time vital sign documentation and sufficient access to computers at intake was also a source of delay in workflows, as staff were required to record the numbers on paper and later transfer them to EPIC, once able to access a shared computer. Having a computer in spaces shared with patients at intake could help facilitate communication, data collection (e.g., real-time documentation of patient BP readings), and patient monitoring.

#### Subtheme 3.2: Technical support demands staff time

Across all sites, staff reported high patient demand for MyChart technical support, which poses significant strain on the administrative staffs' time and effort. Though demand for assistance was described as high across all sites, the degree of hands-on-help available and offered varied by site, depending on site-specific resources (e.g., staffing and time constraints). For example, FQHC staff across all sites reported challenges onboarding patients to MyChart and encouraging engagement. To combat this, many PSAs offered to help patients at appointment checkout by creating or accessing their MyChart account. At some sites, the PSAs went as far as to walk patients through the mobile application with phone in hand; at other sites, PSAs only had enough time to give a flyer or brochure with information. Staff did, however, acknowledge that, since first introducing MyChart, engagement has increased over time, with one PCP remarking that this rise exceeded expectations. This may be attributed to (1) the encouragement offered by PCPs at every clinical appointment and (2) the hands-on support by PSAs in application download, account creation and access (e.g., username and password recovery), and printed resource distribution (e.g., multilingual flyers and business cards), all of which has demanded significant staff time and effort.

### Theme 4 (the CFIR’s inner setting: culture): despite high patient loads, FQHC staff are motivated to provide high-quality care

The CFIR's inner setting domain is defined by the norms, values, and assumptions held by FQHC staff, which may impact implementation quality. Accordingly, as described below, Theme 4 speaks to staff-described values of *recipient-centeredness* (e.g., staff are motivated to serve patients), deliverer-centeredness (e.g., staff recognize that preventing staff stress and burnout is an essential aspect of delivering quality care), and learning-centeredness (e.g., staff are eager to learn, as a means of improving quality care).

#### Subtheme 4.1: Recipient-centeredness

FQHC staff described a culture of patient-centeredness, expressing a profound desire to connect with and assist patients in the long-term. Many FQHC staff stated that they do this work to help patients and support any intervention that advances patient wellness. When asked about their degree of support for incoming interventions (e.g., ALTA), many shared that they were in support of anything that may help the patient. FQHC staff reflected on the impact that an intervention may have on care quality before thinking of how it may affect their own workflow.

#### Subtheme 4.2: Deliverer-centeredness

Adequate FQHC staffing and appropriate scheduling protects FQHC staff against stress and burnout, which, in turn, improves quality of patient care delivered. Reflective of their commitment to patients, FQHC staff relayed concerns regarding the impact understaffing and overscheduling may pose on meeting the needs of rising patient loads. When reflecting on this, respondents reported that having enough staff on the schedule to address patients' needs and give each patient adequate time was essential. Staff also conveyed that to provide patients with the best quality care, they must prioritize their own health and wellness and that of their team members, with whom they shared a sense of commitment and belonging.

#### Subtheme 4.3: Learning-centeredness varies by site

Staff reported a resounding eagerness to learn through, for example, supervised practice, evaluation, or training, as a means of improving patient care. However, the form and frequency of staff evaluation offered by FQHC supervisors, or staff perceptions of evaluation, varied by site. For example, some FQHC staff described thorough evaluation procedures offered by their supervisors, alongside ample training opportunities. Alternatively, at other sites, staff shared they could not recall training opportunities nor formal evaluation opportunities. This was also reflected in BP-specific protocol; some sites demonstrated site-specific trainings, whereas staff at others reported they had not received any training in BP measurement and control since joining the FQHC.

## Discussion

Pre-implementation is a critical period for measuring and identifying key anticipated facilitators and barriers to implementation, especially in complex, high demand environments such as FQHCs; however, methods for doing so are underreported in the literature. The current paper describes the findings of a rigorous pre-implementation evaluation, preceding the implementation of a multi-level intervention targeting hypertension monitoring, medication adherence, and BP control across six primary care sites in a large FQHC delivering high quality care to underserved and immigrant communities of Brooklyn, New York, regardless of ability to pay.

In CFIR’s individual deliverer level, which reflects FQHC staff and leadership readiness for implementation, respondents reported moderate mean adaptive reserve, evidence-based practice attitudes, and implementation leadership scores, which may prove advantageous for ALTA implementation, as has been observed in prior studies reporting comparable scores [43–47]. Specifically, prior studies have linked comparable, moderate scores with greater likelihood of participating in evidence-based interventions [43], implementing best practices [44–46], and reporting a positive implementation climate [47]. Although scores were moderate overall, they did vary by site, indicating that implementers must tailor pre- and mid-implementation supports to site-specific assets and needs, as a means of equitably closing potential implementation gaps. Although fewer than one-in-two staff reported prior training in chronic disease management, patient-centered communication, and quality improvement methods, which potentially represents a barrier to implementation, responsiveness to current training opportunities was high, with nearly all staff having completed assigned modules and a majority passing on the first attempt.

Regarding the CFIR’s individual recipient (patient) needs, a majority of FQHC staff reported both a recognition of and empathy for the barriers patients face in managing their hypertension [13], and consequently expressed a desire to support patients in navigating access to care. This patient-centered perspective and approach to care may facilitate optimal implementation outcomes in hypertension care, as prior literature has demonstrated [48]. However, FQHC staff capacity and self-efficacy to address such needs were limited by staffing capacity, turnover and competing demands, which could serve as another potential barrier to ALTA implementation [49–51]. Further, being that ALTA is a technology-facilitated intervention, and both digital access and literacy are limited within the target population, intervention implementation may fail without proper supports in place [52, 53].

Representative of the CFIR’s innovation domain, FQHC staff recognized an urgent need for improved hypertension care and named patient awareness of BP as a key target of interest, aligning with findings from prior literature [54, 55]. Although most FQHC staff acknowledged pre-existing hypertension workflows, heterogeneity in how much staff adhere to them may present a barrier to implementation [45]. If structures are not in place to equitably train, supervise, and support staff in deploying a novel intervention and integrating it with current practices, then current and novel protocols may be confused, hindering implementation fidelity and adherence. FQHC staff may also feel unmotivated to implement and sustain a novel intervention (e.g., ALTA) if a functional, preexisting hypertension workflow exists [56]. To combat these concerns, ALTA practice facilitators will provide FQHC staff with hands-on supervision and training in the novel workflows and the relative advantage offered by ALTA will be clearly communicated to staff [26].

Reflective of the CFIR’s inner setting domain, FQHC staff reported that limited in-clinic availability of electronic hardware (e.g., computers, tablets) and infrastructure (e.g., Wi-Fi and cell service) may impact workflow, as others have in similar contexts [57–61]. Successful implementation of ALTA¸ a technology-facilitated intervention [26], may require additional investments in digital infrastructure (e.g., equip clinics with computers, tablets, and Wi-Fi), for the overall benefit of clinical workflows [53, 57]. Despite limited resources, however, staff described a patient-centered clinical culture. As such, motivation to implement ALTA was most often attributed to a desire to improve patient outcomes [62]. In line with this patient-centered approach, staff also expressed a clear desire to learn and engage with evaluative processes; responsively, ALTA may offer standardized supervision, evaluation, and training to staff in the pre-implementation and early implementation stages of ALTA, which may positively affect ALTA implementation and overall patient care. Beyond motivation to serve patients, staff also reported a commitment to supporting their fellow coworkers, with whom many identified a sense of “team”, which is critical to the implementation of ALTA’s team-based care approach [32, 63, 64]. Many also reflected on the importance of adequate staffing and scheduling, seen as essential to optimizing performance and preventing burnout and turnover [65–67] Recognizing the need to minimize staff burden, ALTA practice facilitators work with FQHC leadership to integrate ALTA procedures with existing workflow, as a means of reducing staff burden and time per encounter [26].

We must acknowledge limitations to this study. Namely, all data was collected from FQHC leadership and staff, without perspectives from other important ALTA stakeholders (e.g., ALTA practice facilitators and patients), who we do recommend surveying through future research efforts. However, we do triangulate findings using data from multiple sources (e.g., survey, training analytics, and interviews), representing multiple levels (e.g., staff and leadership) and diverse roles (e.g., PCPs, RNs, MAs). We also were able to include a sample size several times larger than most [22], including most staff, in an understudied and underrepresented context of FQHCs. We recognize that social desirability bias may have impacted staff responses, as these surveys and interviews were distributed by and conducted in the context of a workplace; to limit potential bias, staff were given the opportunity to maintain anonymity in their survey submission and interviews were conducted by research staff in a private space [68, 69]. We also acknowledge that due to the cross-sectional nature of data collection, we cannot extrapolate how staff interpretations may have changed over the course of the pre-implementation period, across which, staff responses regarding readiness and motivation to implement ALTA may have shifted; we recommend this as an area for future research in the context of similar interventions.

## Conclusion

Drawing on several rich quantitative and qualitative data sources, this study serves as one of the first to apply the CFIR to the interpretation of a rigorous pre-implementation evaluation within the important context of an FQHC. In doing so, this study offers as a feasible and rapid model of a pre-implementation evaluation methodology, which may be reproduced in similar contexts prior to intervention implementation, allowing implementers the opportunity to acknowledge and address potential facilitators and barriers before implementation rollout. Such pre-implementation evaluations methods may be especially important within the context of FQHCs and primary care settings, where resources are often limited and patient demand for quality services is high.

### Supplementary Information

**Supplementary Material 1.**

## Data Availability

The datasets used and/or analyzed during the current study are available from the corresponding author on reasonable request.

## Abbreviations

FQHC: Federally Qualified Health Center
CFIR: Consolidated Framework for Implementation Research
ALTA: Advancing Medication Adherence for Long-term Improvements in Hypertension through a Team-based Care Approach
REDCap: Research Electronic Data Capture
PCP: Primary care provider
RN: Registered nurse
MA: Medical assistant
PSA: Patient service advocates
SD: Standard deviation
BP: Blood pressure
NYULH: New York University Langone Health
NIMHD: National Institute of Minority Health and Health Disparities

## References

[1] Virani SS, Alonso A, Benjamin EJ, Bittencourt MS, Callaway CW, Carson AP (2020). Heart disease and stroke statistics—2020 update: a report from the American Heart Association. Circulation.

[2] Danaei G, Vander Hoorn S, Lopez AD, Murray CJ, Ezzati M, group CRAc (2005). Causes of cancer in the world: comparative risk assessment of nine behavioural and environmental risk factors. Lancet.

[3] Kearney PM, Whelton M, Reynolds K, Muntner P, Whelton PK, He J (2005). Global burden of hypertension: analysis of worldwide data. Lancet.

[4] Fuchs FD, Whelton PK (2020). High blood pressure and cardiovascular disease. Hypertension.

[5] Bromfield S, Muntner P (2013). High blood pressure: the leading global burden of disease risk factor and the need for worldwide prevention programs. Curr Hypertens Rep.

[6] Havranek EP, Mujahid MS, Barr DA, Blair IV, Cohen MS, Cruz-Flores S (2015). Social determinants of risk and outcomes for cardiovascular disease: a scientific statement from the American Heart Association. Circulation.

[7] Whelton PK, Carey RM, Aronow WS, Casey DE, Collins KJ, Dennison Himmelfarb C (2018). 2017 ACC/AHA/AAPA/ABC/ACPM/AGS/APhA/ASH/ASPC/NMA/PCNA Guideline for the Prevention, Detection, Evaluation, and Management of High Blood Pressure in Adults: executive summary: a report of the american college of cardiology/american heart association task force on clinical practice guidelines. Hypertension.

[8] Centers for Disease Control and Prevention (CDC). In: Hypertension Cascade: Hypertension Prevalence, Treatment and Control Estimates Among US Adults Aged 18 Years and Older Applying the Criteria From the American College of Cardiology and American Heart Association’s 2017 Hypertension Guideline—NHANES 2015–2018. Atlanta, GA: US Department of Health and Human Services; 2021.

[9] Balfour PC, Rodriguez CJ, Ferdinand KC (2015). The role of hypertension in race-ethnic disparities in cardiovascular disease. Curr Cardiovasc Risk Rep.

[10] Kim EJ, Kim T, Paasche-Orlow MK, Rose AJ, Hanchate AD (2017). Disparities in hypertension associated with limited English proficiency. J Gen Intern Med.

[11] Hicken MT, Lee H, Morenoff J, House JS, Williams DR (2014). Racial/ethnic disparities in hypertension prevalence: reconsidering the role of chronic stress. Am J Public Health.

[12] Hill MN, Sutton BS (2000). Barriers to hypertension care and control. Curr Hypertens Rep.

[13] Khatib R, Schwalm J-D, Yusuf S, Haynes RB, McKee M, Khan M (2014). Patient and healthcare provider barriers to hypertension awareness, treatment and follow up: a systematic review and meta-analysis of qualitative and quantitative studies. PLoS One.

[14] Kershaw KN, Khan SS. Moving beyond the individual: multilevel solutions for equitable hypertension control. Circ Cardiovasc Qual Outcome. 2022;15(9):e009374. 10.1161/CIRCOUTCOMES.122.009374 PMC10289055 36065816

[15] Kusuma YS (2010). Migrants’ perceptions on barriers to treatment seeking for hypertension: a qualitative study from Delhi, India. Stud Ethno-Medicine.

[16] Carroll AJ, Mohanty N, Wallace AS, Langman CB, Smith JD (2023). Perspectives of primary care clinicians on the diagnosis and treatment of pediatric hypertension. Fam Community Health.

[17] Stupplebeen DA, Sentell TL, Pirkle CM, Juan B, Barnett-Sherrill AT, Humphry JW (2019). Community health workers in action: community-clinical linkages for diabetes prevention and hypertension management at 3 community health centers. Hawai'i J Med Public Health.

[18] Kasje W, Denig P, Haaijer-Ruskamp F (2002). Specialists’ expectations regarding joint treatment guidelines for primary and secondary care. Int J Qual Health Care.

[19] Payán DD, Sloane DC, Illum J, Vargas RB, Lee D, Galloway-Gilliam L (2017). Catalyzing implementation of evidence-based interventions in safety net settings: a clinical–community partnership in South Los Angeles. Health Promot Pract.

[20] Hyman DJ, Pavlik VN (2000). Self-reported hypertension treatment practices among primary care physicians: blood pressure thresholds, drug choices, and the role of guidelines and evidence-based medicine. Arch Intern Med.

[21] Howes F, Hansen E, Nelson M (2012). Management of hypertension in general practice: a qualitative needs assessment of Australian GPs. Aust Fam Phys.

[22] Taylor SP, Short RT, Asher AM, Taylor B, Beidas RS (2020). A rapid pre-implementation evaluation to inform a family engagement navigator program during COVID-19. Implement Sci Commun.

[23] Ellis J, Band R, Kinsella K, Cheetham-Blake T, James E, Ewings S (2020). Optimising and profiling pre-implementation contexts to create and implement a public health network intervention for tackling loneliness. Implement Sci.

[24] Morris ZS, Wooding S, Grant J (2011). The answer is 17 years, what is the question: understanding time lags in translational research. J R Soc Med.

[25] Damschroder LJ, Reardon CM, Opra Widerquist MA, Lowery J (2022). Conceptualizing outcomes for use with the Consolidated Framework for Implementation Research (CFIR): the CFIR Outcomes Addendum. Implement Sci.

[26] Schoenthaler A, De La Calle F, Soto A, Barrett D, Cruz J, Payano L (2021). Bridging the evidence-to-practice gap: a stepped-wedge cluster randomized controlled trial evaluating practice facilitation as a strategy to accelerate translation of a multi-level adherence intervention into safety net practices. Implement Sci Commun.

[27] Hussey MA, Hughes JP (2007). Design and analysis of stepped wedge cluster randomized trials. Contemp Clin Trials.

[28] Baskerville NB, Liddy C, Hogg W (2012). Systematic review and meta-analysis of practice facilitation within primary care settings. Ann Fam Med.

[29] Goldstein MK, Coleman RW, Tu SW, Shankar RD, O'Connor MJ, Musen MA (2004). Translating research into practice: organizational issues in implementing automated decision support for hypertension in three medical centers. J Am Med Inform Assoc.

[30] Mueller C, Wesenberg S, Nestmann F, Stubbs B, Bebbington P, Raymont V (2018). Interventions to enhance coping after traumatic brain injury: a systematic review. Int J Ther Rehabil.

[31] Ogedegbe G (2008). Barriers to optimal hypertension control. J Clin Hypertens.

[32] Damschroder LJ, Aron DC, Keith RE, Kirsh SR, Alexander JA, Lowery JC (2009). Fostering implementation of health services research findings into practice: a consolidated framework for advancing implementation science. Implement Sci.

[33] Damschroder L, Reardon CM, Widerquist MAO, Lowery JC (2022). The updated consolidated framework for implementation research: CFIR 2.0.

[34] Kirk MA, Kelley C, Yankey N, Birken SA, Abadie B, Damschroder L (2015). A systematic review of the use of the consolidated framework for implementation research. Implement Sci.

[35] Jaén CR, Crabtree BF, Palmer RF, Ferrer RL, Nutting PA, Miller WL (2010). Methods for evaluating practice change toward a patient-centered medical home. Ann Fam Med.

[36] Aarons GA (2004). Mental health provider attitudes toward adoption of evidence-based practice: the evidence-based practice attitude scale (EBPAS). Ment Health Serv Res.

[37] Aarons GA, Ehrhart MG, Farahnak LR (2014). The implementation leadership scale (ILS): development of a brief measure of unit level implementation leadership. Implement Sci.

[38] Target:BP. TOOLS & DOWNLOADS: American Heart Association; Available from: https://targetbp.org/tools-downloads/?sort=topic&. Accessed 1 May 2023.

[39] Chobanian AV, Bakris GL, Black HR, Cushman WC, Green LA, Izzo JL (2003). The seventh report of the joint national committee on prevention, detection, evaluation, and treatment of high blood pressure: the JNC 7 report. JAMA.

[40] Bryman A, Burgess RG (1994). Developments in qualitative data analysis: an introduction. Analyzing qualitative data.

[41] McDonald N, Schoenebeck S, Forte A. Reliability and inter-rater reliability in qualitative research: Norms and guidelines for CSCW and HCI practice. Proceedings of the ACM on Human-Computer Interaction. 2019;3(CSCW):1–23.

[42] Dedoose Version 9.0.17, web application for managing, analyzing, and presenting qualitative and mixed method research data. Los Angeles, CA: SocioCultural Research Consultants, LLC; 2021. www.dedoose.com

[43] Sohng HY, Kuniyuki A, Edelson J, Weir RC, Song H, Tu S-P (2013). Capability for change at community health centers serving Asian Pacific Islanders: an exploratory study of a cancer screening evidence-based intervention. Asian Pac J Cancer Prev.

[44] Tu SP, Young VM, Coombs LJ, Williams RS, Kegler MC, Kimura AT (2015). Practice adaptive reserve and colorectal cancer screening best practices at community health center clinics in 7 states. Cancer.

[45] Platis C, Delimpaltadakis E, Stergiannis P, Kostagiolas P, Intas G, editors. Evidence-Based Leadership: A Study of Its Application to General Hospital of the Public Health System Through the Implementation Leadership Scale. GeNeDis 2020: Geriatrics: Springer; 2021. 10.1007/978-3-030-78771-4_2 34972886

[46] Stokke K, Olsen NR, Espehaug B, Nortvedt MW (2014). Evidence based practice beliefs and implementation among nurses: a cross-sectional study. BMC Nurs.

[47] Williams NJ, Wolk CB, Becker-Haimes EM, Beidas RS (2020). Testing a theory of strategic implementation leadership, implementation climate, and clinicians’ use of evidence-based practice: a 5-year panel analysis. Implement Sci.

[48] Buawangpong N, Pinyopornpanish K, Jiraporncharoen W, Dejkriengkraikul N, Sagulkoo P, Pateekhum C (2020). Incorporating the patient-centered approach into clinical practice helps improve quality of care in cases of hypertension: a retrospective cohort study. BMC Fam Pract.

[49] Woltmann EM, Whitley R, McHugo GJ, Brunette M, Torrey WC, Coots L (2008). The role of staff turnover in the implementation of evidence-based practices in mental health care. Psychiatr Serv.

[50] Bettencourt AF, Gross D, Breitenstein S (2019). Evaluating implementation fidelity of a school-based parenting program for low-income families. J Sch Nurs.

[51] Locke J, Olsen A, Wideman R, Downey MM, Kretzmann M, Kasari C (2015). A tangled web: The challenges of implementing an evidence-based social engagement intervention for children with autism in urban public school settings. Behav Ther.

[52] Band R, Bradbury K, Morton K, May C, Michie S, Mair FS (2017). Intervention planning for a digital intervention for self-management of hypertension: a theory-, evidence-and person-based approach. Implement Sci.

[53] Palacholla RS, Fischer N, Coleman A, Agboola S, Kirley K, Felsted J (2019). Provider-and patient-related barriers to and facilitators of digital health technology adoption for hypertension management: scoping review. JMIR cardio.

[54] Kaboli PJ, Shivapour DM, Henderson MS, Barnett MJ, Ishani A, Carter BL (2007). Patient and provider perceptions of hypertension treatment: do they agree?. J Clin Hypertens.

[55] Roumie CL, Greevy R, Wallston KA, Elasy TA, Kaltenbach L, Kotter K (2011). Patient centered primary care is associated with patient hypertension medication adherence. J Behav Med.

[56] Helmink JH, Kremers SP, van Boekel LC, van Brussel-Visser FN, de Vries NK (2012). Factors determining the motivation of primary health care professionals to implement and continue the ‘Beweegkuur’lifestyle intervention programme. J Eval Clin Pract.

[57] Payán DD, Frehn JL, Garcia L, Tierney AA, Rodriguez HP (2022). Telemedicine implementation and use in community health centers during COVID-19: clinic personnel and patient perspectives. SSM-Qual Res Health.

[58] Chen AH, Kushel MB, Grumbach K, Yee HF (2010). A safety-net system gains efficiencies through ‘eReferrals’ to specialists. Health Aff.

[59] Kim Y, Chen AH, Keith E, Yee HF, Kushel MB (2009). Not perfect, but better: primary care providers’ experiences with electronic referrals in a safety net health system. J Gen Intern Med.

[60] Fort MP, Namba LM, Dutcher S, Copeland T, Bermingham N, Fellenz C (2017). Implementation and evaluation of the safety net specialty care program in the Denver metropolitan area. Perm J.

[61] Nakamura Y, Laberge M, Davis A, Formoso A (2019). Barriers and strategies for specialty care access through federally qualified health centers: a scoping review. J Health Care Poor Underserved.

[62] Légaré F, Ratté S, Gravel K, Graham ID (2008). Barriers and facilitators to implementing shared decision-making in clinical practice: update of a systematic review of health professionals’ perceptions. Patient Educ Couns.

[63] Proia KK, Thota AB, Njie GJ, Finnie RK, Hopkins DP, Mukhtar Q (2014). Team-based care and improved blood pressure control: a community guide systematic review. Am J Prev Med.

[64] Edmondson AC, Bohmer RM, Pisano GP (2001). Disrupted routines: Team learning and new technology implementation in hospitals. Adm Sci Q.

[65] Linzer M, Levine R, Meltzer D, Poplau S, Warde C, West CP (2014). 10 bold steps to prevent burnout in general internal medicine. J Gen Intern Med.

[66] Levine S, Unützer J, Yip JY, Hoffing M, Leung M, Fan M-Y (2005). Physicians' satisfaction with a collaborative disease management program for late-life depression in primary care. Gen Hosp Psychiatry.

[67] Misra-Hebert AD, Perzynski A, Rothberg MB, Fox J, Mercer MB, Liu X (2018). Implementing team-based primary care models: a mixed-methods comparative case study in a large, integrated health care system. J Gen Intern Med.

[68] Donaldson SI, Grant-Vallone EJ (2002). Understanding self-report bias in organizational behavior research. J Bus Psychol.

[69] Bergen N, Labonté R (2020). “Everything is perfect, and we have no problems”: detecting and limiting social desirability bias in qualitative research. Qual Health Res.

