# Supplementary material for: “Hypertension is such a difficult disease to manage”: federally qualified health center staff- and leadership-perceived readiness to implement a technology-facilitated team-based hypertension model
Source: Implement Sci Commun. 2024 May 2;5:49. doi: 10.1186/s43058-024-00587-8 (PMC11067286; doi:10.1186/s43058-024-00587-8)
Supplement: Supplementary file 1 — Supplementary Material 1. [file 43058_2024_587_MOESM1_ESM.docx]

**SUPPLEMENTS**

**Supplemental Table 1. FQHC staff and leadership response rates (eligible vs. participated) by measure (2021-2022)**

|  | | **Eligible** ^d^ | **Participated** | |
| --- | --- | --- | --- | --- |
| **Surveys** | | n | n | % ^e^ |
|  | Leadership | 23 | 18 | 78.3 |
|  | PCPs ^a^ | 44 | 32 | 72.7 |
|  | RNs | 18 | 12 | 66.7 |
|  | MAs | 44 | 30 | 68.2 |
|  | Other ^b^ | 47 | 32 | 68.1 |
|  | **Total** | **176** | **124** | **70.5** |
| **Interviews** | |  |  |  |
|  | PCPs ^a^ | 31 | 8 | 25.8 |
|  | RNs | 17 | 11 | 64.7 |
|  | MAs | 44 | 28 | 63.6 |
|  | PSAs | 46 | 25 | 54.3 |
|  | **Total** | **138** | **67** | **48.6** |
| **Training 1: Accurate blood pressure measurement in office** ^c^ | |  |  |  |
|  | RNs | 22 | 19 | 86.4 |
|  | MAs | 46 | 46 | 100.0 |
|  | Other ^b^ | 5 | 4 | 80.0 |
|  | **Total** | **73** | **69** | **94.5** |
| **Training 2: Self-measured blood pressure** ^c^ | |  |  |  |
|  | RNs | 19 | 18 | 94.7 |
|  | MAs | 6 | 6 | 100.0 |
|  | Other ^b^ | 7 | 5 | 71.4 |
|  | **Total** | **32** | **29** | **90.6** |
| **Training 3: Quality improvement** ^c^ | |  |  |  |
|  | RNs | 11 | 10 | 90.9 |
|  | MAs | 15 | 13 | 86.7 |
|  | Other ^b^ | 5 | 5 | 100.0 |
|  | **Total** | **31** | **28** | **90.3** |

PCP = primary care physician (including physicians, physicians assistants, nurse practitioners, and licensed practical nurses), RN = registered nurse, MA= medical assistant, and PSA = patient services advocate.

^a^ Residents were eligible for surveys, but no interviews, accounting for the difference in number eligible for surveys vs. interviews.

^b^ Examples of roles in the “other” category include site coordinators, clerks, and PSAs.

^c^ Those assigned to these training courses at the discretion of their supervisor are considered “eligible”.

^d^ Note that the number of eligible respondents may differ across measures due to FQHC staffing changes across the time points in which measures were administered.

^e^ Row percent presented.

**Supplemental Table 2. Mean adaptive reserve scores and frequency of item responses (n=124)**

|  |  |  | **Overall**  **(n=124)** ^a^ | **Minimum across sites** ^b^ | **Maximum across sites** ^b^ |
| --- | --- | --- | --- | --- | --- |
| **Summary score** ^c^ | | | **Mean (SD)** | **Mean (SD)** | **Mean (SD)** |
|  | Adaptive reserve score (range: 0-1) | | 0.7 (0.1) | 0.7 (0.2) | 0.8 (0.2) |
| **Frequency agree or strongly agree by item** ^d^ | | | **%** | **%** | **%** |
|  | 1. Mistakes have led to positive changes here | | 65.3 | 55.0 | 80.0 |
|  | 2. I have many opportunities to grow in my work | | 65.3 | 40.0 | 80.0 |
|  | 3. People in our practice actively seek new ways to improve how we do things | | 70.2 | 50.0 | 90.0 |
|  | 4. People at all levels of this office openly talk about what is and isn't working | | 62.9 | 50.0 | 69.2 |
|  | 5. Leadership strongly supports practice change efforts | | 68.6 | 57.2 | 76.9 |
|  | 6. After trying something new, we take time to think about how it worked | | 66.1 | 50.0 | 73.1 |
|  | 7. Most of the people who work in our practice seem to enjoy their work | | 66.1 | 45.0 | 77.1 |
|  | 8. It is hard to get things to change in our practice. (Reverse) ^e^ | | 38.7 | 30.0 | 48.6 |
|  | 9. This practice is a place of joy and hope | | 57.3 | 40.0 | 69.2 |
|  | 10. This practice learns from its mistakes | | 65.3 | 50.0 | 76.9 |
|  | 11. Practice leadership promotes an environment that is an enjoyable place to work | | 63.7 | 50.0 | 73.1 |
|  | 12. People in this practice operate as a real team | | 65.3 | 38.1 | 90.0 |
|  | 13. When we experience a problem in the practice, we make a serious effort to figure out what's really going on | | 69.4 | 58.3 | 74.3 |
|  | 14. Leadership in this practice creates an environment where things can be accomplished | | 67.7 | 57.2 | 73.1 |

^a^ All FQHC leadership and FQHC staff (n=176) were asked to complete this scale as part of the survey; of those eligible, 70.4% (n=124) completed the scale.

^b^ Site-level mean (SD) score and frequencies are compared; both the minimum and the maximum observed across sites are presented.

^c^ Adaptive reserve score is calculated as a mean of responses across all items (n=14, range=0-1) (35)

^d^ Presented are the percentage of respondents who agree or strongly agree with each item.

^e^ Higher frequency agreement for item 8 is interpreted as disadvantageous; item 8 is reverse coded in the calculation of the adaptive reserve score.

**Supplemental Table 3. Mean evidence-based practice scores and frequency of item responses (n=106)**

|  |  |  | **Overall**  **(n=106)** ^a^ | **Minimum across sites** ^b^ | **Maximum across sites** ^b^ |
| --- | --- | --- | --- | --- | --- |
| **Summary scores** ^c^ | | | **Mean (SD)** | **Mean (SD)** | **Mean (SD)** |
|  | Evidence-based practice attitudes score (range: 0-4) | | 2.7 (0.7) | 2.3 (0.7) | 3.1 (0.7) |
|  | *Openness subscore (range: 0-4)* | | 2.8 (0.9) | 2.1 (1.1) | 3.2 (0.8) |
|  | *Divergence subscore (range: 0-4; reverse)* ^e^ | | 1.8 (1.0) | 1.4 (0.9) | 2.3 (0.8) |
|  | *Appeal subscore (range: 0-4)* | | 2.6 (0.9) | 2.2 (0.9) | 2.9 (0.8) |
|  | *Requirements subscore (range: 0-4)* | | 2.6 (1.0) | 1.8 (0.9) | 3.0 (0.8) |
|  |  | |  |  |  |
| **Frequency agree to a great or very great extent by item** | | | **%** | **%** | **%** |
| **Openness** | | |  |  |  |
|  | 1. I like to use new types of interventions to help my patients | | 54.8 | 30.0 | 69.2 |
|  | 2. I am willing to try new types of interventions even if I have to follow a treatment manual | | 58.1 | 20.0 | 71.4 |
|  | 3. I am willing to use new and different types of interventions developed by researchers | | 56.5 | 20.0 | 69.2 |
| **Divergence** | | |  |  |  |
|  | 4. Research based interventions are not clinically useful (Reverse) ^e^ | | 23.4 | 10.0 | 50.0 |
|  | 5. Clinical experience is more important than using manualized interventions (Reverse) ^e^ | | 27.4 | 10.0 | 41.7 |
| **Appeal** | | |  |  |  |
|  | 6. Likely to a new intervention that “made sense” to you | | 47.6 | 28.6 | 58.3 |
|  | 10. Likely to adopt a new intervention that was being used by colleagues who were happy with it | | 43.6 | 28.6 | 66.7 |
|  | 11. Likely to adopt a new intervention you felt you had enough training to use correctly | | 51.6 | 28.6 | 75.0 |
| **Requirements** | | |  |  |  |
|  | 7. Likely to adopt a new intervention that was required by your supervisor | | 44.4 | 10.0 | 58.3 |
|  | 8. Likely to adopt a new intervention that was required by your clinic | | 42.7 | 10.0 | 58.3 |
|  | 9. Likely to adopt a new intervention that was required by your state | | 46.0 | 20.0 | 58.3 |

^a^ All FQHC leadership and FQHC staff (n=153) were asked to complete this scale as part of the survey; of those eligible, 69.3% (n=106) completed the scale.

^b^ Site-level mean (SD) score and frequencies are compared; both the minimum and the maximum observed across sites are presented.

^c^ Evidence-based practice attitudes score is calculated as a mean of responses across all items (n=9, range=0-4) (36).

^d^ Presented are the percent of respondents who agree or strongly agree with each item.

^e^ Higher diverge score and higher frequency agreement items 4 and 5 are interpreted as disadvantageous; items 4 and 5 are reverse coded in the calculation of the evidence-based practice score**.**

**Table S4. Mean implementation leadership scores and frequency of item responses (n=124)**

|  |  |  | **Overall**  **(n=124)** ^a^ | **Minimum across sites** ^b^ | **Maximum across sites** ^b^ |
| --- | --- | --- | --- | --- | --- |
| **Summary score** ^c^ | | | **Mean (SD)** | **Mean (SD)** | **Mean (SD)** |
|  | Implementation leadership (range: 0-4) | | 2.5 (0.9) | 2.0 (1.1) | 2.9 (0.9) |
|  | *Knowledgeable subscore (range: 0-4)* | | 2.6 (1.0) | 2.3 (0.5) | 3.0 (1.1) |
|  | *Supportive subscore (range: 0-4)* | | 2.7 (1.0) | 2.1 (1.2) | 3.0 (1.0) |
|  | *Perseverant subscore (range: 0-4)* | | 2.6 (1.0) | 2.0 (1.2) | 2.9 (0.9) |
|  | *Proactive subscore (range: 0-4)* | | 2.4 (1.1) | 1.9 (1.1) | 2.7 (1.2) |
|  |  | |  |  |  |
| **Frequency agree or strongly agree by item** ^d^ | | | **%** | **%** | **%** |
| *Asked of FQHC staff and leadership: To what extent do you agree that the medical director…* | | |  |  |  |
| **Knowledgeable leadership** | | |  |  |  |
|  | 1. Knows about evidence-based practice for hypertension management | | 53.2 | 30.0 | 66.7 |
|  | 2. Recognizes and appreciates FQHC staffs' efforts toward successful implementation of evidence-based practice for hypertension management | | 57.3 | 40.0 | 66.7 |
| **Supportive leadership** | | |  |  |  |
|  | 3. Supports FQHC staffs' efforts to learn more about evidence-based practice for hypertension management | | 62.9 | 50.0 | 75.0 |
|  | 4. Supports FQHC staffs' efforts to use evidence-based practice for hypertension management | | 62.9 | 40.0 | 75.0 |
| **Perseverant leadership** | | |  |  |  |
|  | 5. Carries on through the challenges of implementing evidence-based practice for improving hypertension management | | 56.5 | 40.0 | 66.7 |
|  | 6. Reacts to critical issues regarding the implementation of evidence-based practice for improving hypertension management by openly and effectively addressing the problem(s) | | 57.3 | 40.0 | 66.7 |
| **Proactive leadership** | | |  |  |  |
|  | 7. Removed obstacles to the implementation of evidence-based practice for improved hypertension management | | 42.8 | 10.0 | 61.5 |
|  | 8. Established clear standards for the implementation of evidence-based practice for improved hypertension management | | 49.2 | 20.0 | 66.7 |
| *Asked of FQHC leadership only: To what extent do you agree that you…* | | |  |  |  |
|  | 9. Developed a plan to facilitate implementation  of evidence-based practice for hypertension  management | | 10.0 | N/A ^e^ | 10.0 |

^a^ All FQHC leadership and FQHC staff (n=176) were asked to complete this scale as part of the survey; of those eligible, 70.4% (n=124) completed the scale

^b^ Site-level mean (SD) score and frequencies are compared; both the minimum and the maximum observed across sites are presented

^c^ Implementation leadership score is calculated as a mean of responses across all items (n=8 for FQHC staff and n=9 for leadership, range=0-4) (37)

^d^ Presented are the percent of respondents who agree or strongly agree with each item

^e^ Item 9 is only asked of FQHC leadership
